# Evaluation of Polyvinyl Alcohol as Binder during Continuous Twin Screw Wet Granulation

**DOI:** 10.3390/pharmaceutics16070854

**Published:** 2024-06-25

**Authors:** Phaedra Denduyver, Gudrun Birk, Alessandra Ambruosi, Chris Vervaet, Valérie Vanhoorne

**Affiliations:** 1 Laboratory of Pharmaceutical Technology, Department of Pharmaceutics, Ghent University, Ottergemsesteenweg 460, 9000 Ghent, Belgium; phaedra.denduyver@ugent.be (P.D.); chris.vervaet@ugent.be (C.V.); 2 Merck KGaA, Frankfuter Str. 250, 64293 Darmstadt, Germany; gudrun.birk@merckgroup.com (G.B.); alessandra.ambruosi@merckgroup.com (A.A.)

**Keywords:** continuous manufacturing, twin-screw granulation, wet granulation, polyvinyl alcohol, formulation optimization, binders

## Abstract

Binder selection is a crucial step in continuous twin-screw wet granulation (TSWG), as the material experiences a much shorter residence time (2–40 s) in the granulator barrel compared to batch-wise granulation processes. Polyvinyl alcohol (PVA) 4-88 was identified as an effective binder during TSWG, but the potential of other PVA grades—differing in polymerization and hydrolysis degree—has not yet been studied. Therefore, the aim of the current study was to evaluate the potential of different PVA grades as a binder during TSWG. The breakage and drying behavior during the fluidized bed drying of drug-loaded granules containing the PVA grades was also studied. Three PVA grades (4-88, 18-88, and 40-88) were characterized and their attributes were compared to previously investigated binders by Vandevivere et al. through principal component analysis. Three binder clusters could be distinguished according to their attributes, whereby each cluster contained a PVA grade and a previously investigated binder. PVA 4-88 was the most effective binder of the PVA grades for both a good water-soluble and water-insoluble formulation. This could be attributed to its high total surface energy, low viscosity, good wettability of hydrophilic and hydrophobic surfaces, and good wettability by water of the binder. Compared to the previously investigated binders, all PVA grades were more effective in the water-insoluble formulation, as they yielded strong granules (friability below 30%) at lower L/S-ratios. This was linked to the high dispersive surface energy of the high-energy sites on the surface of PVA grades and their low surface tension. During fluidized bed drying, PVA grades proved suitable binders, as the acetaminophen (APAP) granules were dried within a short time due to the low L/S-ratio, at which high-quality granules could be produced. In addition, no attrition occurred, and strong tablets were obtained. Based on this study, PVA could be the preferred binder during twin screw granulation due to its high binder effectiveness at a low L/S-ratio, allowing efficient downstream processing. However, process robustness must be controlled by the included excipients, as PVA grades are operating in a narrow L/S-ratio range.

## 1. Introduction

Granulation remains an essential manufacturing technology in the production of oral solid dosage forms since the majority of drug-loaded formulations are unsuitable for direct compression due to limited flowability and compactibility [1,2]. Recently, twin-screw wet granulation (TSWG) has become the preferred granulation technique, as it can be implemented into a fully continuous from-powder-to-tablet line [3]. Continuous manufacturing processes can yield high-quality products by the implementation of process analytical technology, the elimination of scale-up, and minimizing product variability [3,4,5]. TSWG is a flexible process due to the use of a modular screw configuration and its continuous nature [6]. Compared to batch manufacturing, the residence time of materials in the twinscrew granulator (TSG) is short (2–40 s); hence, a fast interaction between the solids and liquid phase is needed [7]. Therefore, the composition of a formulation processed via TSWG should be approached differently compared to batch granulation, as it is key to yield high-quality granules via continuous granulation.

Typically, binders are included in the formulation to enhance the bonds formed between the powder particles during granule growth [8]. Most studies investigating binders during TSWG focused on the impact of the binder viscosity on granule quality [9,10,11] or studied a binder type in different formulations hampering the systematic comparison of binders [5,12,13]. Therefore, Vandevivere et al. identified critical binder attributes impacting the granule quality of two standard formulations with different solubility. The formulation consisted of 95% *w*/*w* filler and 5% *w*/*w* binder, using a good water-soluble filler (mannitol) as well as a water-insoluble filler (dicalcium phosphate (DCP)). The binder effectiveness (i.e., liquid-to-solid ratio (L/S-ratio) needed to achieve a granule friability < 30%) was correlated to 15 binder attributes, including properties such as particle size, dissolution rate, wettability, surface tension, and viscosity [14,15]. For the water-insoluble formulation, polyvinyl alcohol (PVA) 4-88 and starch octenyl succinate CO 01 (SOS CO 01) were identified as the most effective binders, as they produced the strongest granules (i.e., granules with the lowest friability) at the lowest L/S-ratios. Binder attributes resulting in a high binder effectiveness were a good wettability of the powder bed as well as easy wetting of the binder with water. Additionally, a high binder viscosity and low surface tension positively influenced binder effectiveness of the other binders [14]. In the good water-soluble formulation, efficient binders were characterized by the good wetting properties of mannitol, fast binder wetting by water, low viscosity, and fast dissolution kinetics. These binders were hydroxypropyl (HP) pea starch, maltodextrin 6, and polyvinyl pyrrolidone (PVP) K12. PVA 4-88 and SOS CO 01 were less effective but yielded the strongest granules irrespective of the L/S-ratio [15]. In a follow-up study, Vandevivere et al. [16] investigated the effect of the binder type (HP pea starch, hydroxypropyl methylcellulose (HPMC) E15, PVP K12, and SOS CO 01) on the granule-drying process and granule breakage behavior in a semi-continuous fluid bed dryer integrated in the ConsiGma^TM^-system. The drying conditions depended on the binder type and L/S-ratio. No effect of the binder type on the granule breakage during granulation followed by the gravimetric transfer of the wet granules to a fluid bed dryer was observed. Although Vandevivere et al. concluded that PVA 4-88 was a suitable binder for the mannitol- and DCP-based formulations, PVA was not included in the study to investigate the impact of binders on the drying efficiency and granule breakage during the fluidized-bed drying (FBD) of acetaminophen (APAP) granules [16].

As PVA 4-88 was an effective binder at a low L/S-ratio during wet granulation, this could shorten the drying time, which can be a limiting factor for the throughput of a continuous process [17]. In addition, PVA is available in different grades, which could aid formulation development by selecting a PVA grade with appropriate attributes for the specific formulation. The grades differ in molecular weight and residual content of acetyl groups. The first nomenclature number of a PVA grade refers to its viscosity (mPa.s, of a 4% aqueous solution at 20 °C), determined by the molecular weight and controlled by the degree of polymerization (DP). The second nomenclature number reflects the degree 9 of hydrolysis (DH), as polyvinyl alcohol is formed by hydrolyzing polyvinyl acetate in ethanol with potassium hydroxide. DP and DH determine the properties of PVA in water, such as solubility, viscosity, wettability and surface tension [18]. Therefore, the current study aimed at gaining an in-depth understanding of the critical binder attributes for the production of high-quality granules at a low L/S-ratio for formulations with diverse properties in terms of solubility by evaluating the potential of different PVA grades as binder during TSWG. PVA grades 4-88, 18-88, and 40-88 were characterized, and their binder attributes were linked to the binder effectiveness as well as the lowest achievable friability during the TSWG of formulations with different solubility (i.e., mannitol- and DCP-based). Next, a comparison was made between the performance of the PVA grades and the previously investigated binders by Vandevivere et al. Finally, the drying efficiency and breakage behavior during FBD of APAP granules produced with different PVA grades were investigated, followed by studying their tabletability.

## 2. Materials and Methods

### 2.1. Materials

Three PVA grades were studied as TSWG binders: PVA 4-88 (Parteck^®^ MXP, Merck, Darmstadt, Germany), PVA 18-88 (Merck, Darmstadt, Germany), and PVA 40-88 (Parteck^®^ SRP 80, Merck, Darmstadt, Germany). Anhydrous DCP (Calipharm^®^ A, Innophos, Chicago Heights, IL, USA) and mannitol (Pearlitol^®^ 50C, Roquette Frères, Lestrem, France) were used as water-insoluble and water-soluble fillers, respectively. Paracetamol Semi-Fine Powder (Mallinckrodt, Hazelwood, MI, USA) was used as a sparingly water-soluble model drug. Magnesium stearate (Ligamed MF-2-V, Peter Greven, Bad Münstereifel, Germany) was used as a lubricant during tableting. A comparison was made with other binders studied by our research group: PVA 4-88 (Parteck^®^ MXP, Merck, Darmstadt, Germany), SOS CO 01 (Cleargum^®^ CO 01, Roquette Frères, Lestrem, France), PVP K90 (BASF, Ludwigshafen, Germany), and HPMC E15 (Methocel^®^ E15, Dow Chemical Company, Rheinmünster, Germany) [14,15,16].

### 2.2. Methods for Binder Characterization

Binders were characterized by particle size, dissolution kinetics, wettability, surface tension, viscosity, and surface energy. The characterization data of SOS CO 01, HP pea starch, maltodextrin 6, PVP K12, PVP K30, PVP K90, and HPMC E15 were used from previous studies of our research group, whereas the PVA grades were characterized using identical characterization procedures during the current study [14,15]. Inverse gas chromatography was additionally performed on SOS CO 01, PVP K90, and HPMC E15 in the current study.

#### 2.2.1. Particle Size Analysis

The particle size distribution (PSD) of the binders was measured in triplicate by laser diffraction (Malvern Mastersizer 3000, Malvern Instruments, Worcestershire, UK). The measurements were performed via the dry dispersion method in volumetric distribution mode. Prior to the measurement, pressure tritation was performed to determine the pressure required to measure the primary particle size of the binders, i.e., a pressure which disperses agglomerates into primary particles without shattering the primary particles. The dry dispersion unit was set at a specific feed rate to ensure an obscuration ranging between 0.5 and 8%. The 10%, 50%, and 90% cumulative undersize fractions of the volume distribution were reported as Dv_10, Dv_50, and Dv_90.

#### 2.2.2. Dissolution Kinetics

The dissolution rate of the binders in demineralized water was characterized by measuring the refractive index. First, a calibration curve was set up by measuring the refractive index of five aqueous binder solutions with different binder concentrations ranging from 1 to 10% *w*/*w*. Then, the binder (10% *w*/*w*) was added to demineralized water and mixed for 30, 60, or 90 s (Bamix mixer, Mettlen, Switzerland). Finally, the solution was filtered using a cellulose-based filter with an 8 μm pore size (Grade 2, Whatman, MA, USA), and the concentration was calculated based on the determined refractive index of the filtered solution using a refractometer (Carl Zeiss, Jena, Germany). Measurements were performed in triplicate. The dissolved binder fractions measured after 30, 60, and 90 s were reported as DissRate_30, DissRate_60, DissRate_90, respectively.

#### 2.2.3. Wettability

The wettability of the binders was determined via contact angle measurements, applying the sessile drop method on the Drop Shape Analyzer (DSA 30, KRÜSS, Hamburg, Germany). Several types of measurements were executed: (i) water on a binder tablet (CAbinder_t0 and CAbinder_t30), (ii) binder solution (8% *w*/*w*) on a DCP tablet (CADCP_t0), and (iii) binder solution (8% *w*/*w*) on a mannitol tablet (CAmannitol_t0 and CAmannitol_t30). A 5 µl droplet was placed on the tablet, and the contact angle (°) was measured immediately (t0) and after 30 s (t30). Since DCP tablets have high porosity, the measurements for (ii) were only performed on timepoint 0 s. Measurements were performed in triplicate. Binder and mannitol tablets were made using a hydraulic press (Specac pellet press, Kent, UK) with a 13 mm diameter die at a pressure of 98 kN for 1 min. DCP was compressed with a compaction simulator (Styl’One Evoluation, Medelpharm, Lyon, France) with a 10 mm diameter punch at a pressure of 25 kN. DCP tablets with high porosity were obtained since the tableting of pure DCP resulted in high ejection forces and no higher forces could be applied.

#### 2.2.4. Surface Tension

The surface tension was measured applying the pendant drop method in air using the Drop Shape Analyzer (DSA 30, KRÜSS, Hamburg, Germany). Aqueous solutions of the binders (8% *w*/*w*) were prepared and their density (m/V) was determined. A liquid droplet of maximum volume was formed at the tip of a polytetrafluoroethylene needle with a diameter of 2 mm. Subsequently, the surface tension was calculated using the curvature of the drop profile [19]. Measurements were performed in triplicate, and the surface tension was reported as ST.

#### 2.2.5. Viscosity

The dynamic viscosity of the aqueous binder solutions was determined using a rotational rheometer (HAAKE MARS^®^ III, Thermo Scientific, Boston, MA, USA). The plate–plate technique was applied, and binder solutions were held at a temperature of 25 °C. Each binder was measured at a concentration of 8% *w*/*w*. Additionally, PVA 4-88, PVA 18-88, and PVA 40-88 were measured at concentrations of 14 and 20% *w*/*w*, 6 and 10% *w*/*w*, and 2 and 4% *w*/*w*, respectively, covering the concentration range, yielding low to highly viscous solutions. Of the sample, 5 mL was loaded between two plates, whereby the position of the upper plate was manually controlled. The upper plate determines the initial gap size, which needs to provide good contact between the measuring geometry and the sample (gap range: 0.9–1.1 mm). The shear rate ranged from 0.1 to 1000 s^−1^, collecting 20 data points. The viscosity values (mPa.s) were derived from a shear rate range (20.7 to 143.8 s^−1^), at which the binder solutions showed Newtonian behavior. The data were corrected by the rheometer software for the non-homogeneous flow inherent to the plate–plate geometry. The viscosity of the 8% *w*/*w* binder solutions was reported as the DynamicViscosity. Furthermore, the slope of the linear correlation between the concentration of binder solution and the average logarithmic viscosity was reported as the ViscositySlope.

#### 2.2.6. Inverse Gas Chromatography

Inverse gas chromatography was conducted to measure the surface energy of the binders using the Surface Energy Analyzer (SEA, Surface Measurement Systems, Alperton, UK). Samples were packed into silanized glass columns (300 mm length × 3–4 mm inner diameter), using silanized glass wool to seal the powder sample. Specific surface area measurements were performed to ensure a standardized amount of the sample was packed in the silanized glass column. Nitrogen was used as carrier gas at a flow rate of 10 mL/min. Analysis was carried out at 30 °C and 0% RH, and samples were preconditioned for 120 min. Series of alkanes and polar organic solvents were used as probes to determine the dispersive (*n*-hexane, *n*-heptane, *n*-octane, *n*-nonane) and specific (ethyl acetate, chloroform) surface energies, respectively. The solvent injections were controlled to achieve probe surface coverages in the range of 0.01 to 0.20 fractional coverage. Due to the gradual increase in the injected vapor, a surface energy heterogeneity plot was generated. The dispersive and specific parts of the surface energy were determined using the Dorris and Gray method and Della Volpe scale, respectively [20,21]. The total surface energy was calculated as the sum of the dispersive and specific parts.

### 2.3. Principal Component Analysis

Principal component analysis (PCA) was performed on the binder characteristics of the PVA grades and previously investigated binders using the SIMCA^®^ 16 software (Sartorius Stedium Biotech, Umeå, Sweden). The raw data of the binder characterization are shown in Table A1. Data were mean centered and scaled to unit variance. A logarithmical transformation was performed on the DynamicViscosity values to make the data symmetrically distributed. Block scaling was applied to the laser diffraction and dissolution parameters resulting in a variance of the block equaling 1. Hierarchical cluster analysis (calculated with Ward’s method and sorted by size [22]) was applied to define binder clusters.

### 2.4. Preparation of Granules

**Figure 1:**
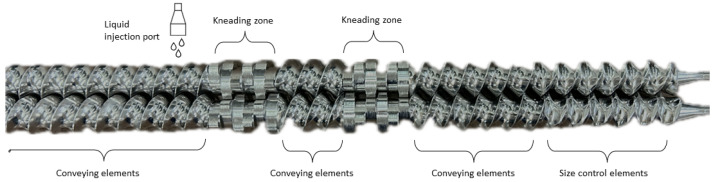
Screw configuration with material flow from left to right. Kneading zones consist of 6 kneading elements (length-to-diameter (L/D) 1/4) in a stagger angle of 60°. Granulation liquid was added just before the first kneading zone.

The water-soluble and water-insoluble formulations were granulated on the ConsiGmaTM-25 system (C25). DCP or mannitol (95% *w*/*w*) and PVA grades (5% *w*/*w*) were preblended in a 20 L tumbling mixer (Inversina Bioengineering, Wald, Switzerland) at 25 rotations per minute (rpm) for 15 min. The preblends were transferred to the loss-in-weight feeder (KT20, K-Tron Soder, Niederlenz, Switzerland), and subsequently fed into the granulation unit at a throughput of 20 kg/h. The granulation unit consisted of two co-rotating screws with a length-to-diameter ratio of 20/1. The screws consisted of two kneading zones with each of the six kneading elements placed at a stagger angle of 60°, separated by conveying elements (Figure 1). At the end of the screws, size control elements were positioned to break up the oversized granules. The screw speed was set at 300 and 500 rpm for DCP and mannitol, respectively. Demineralized water as a granulation liquid was added just before the first kneading zone via the loss-in-weight principle using two silicon tubings (1.6 mm internal diameter) connected with two 1.6 mm nozzles and two out-of-phase peristaltic pumps (Watson Marlow, Cornwall, UK). The L/S-ratio range was preliminarily examined, and granules were collected over a L/S-ratio range, which yielded a large fines fraction at the low end and oversized granules at the high end (Table 1). Temperature was kept constant at 30 °C by an active cooling system of the granulator barrel. The torque was monitored by a built-in torque gauge at 1 s intervals (and with a resolution of 0.1 Nm). After stabilization of the torque, 1000 g granules were collected and subsequently tray-dried in an oven at 40 °C until a loss-on-drying (LOD) value between 1 and 3% was obtained. The moisture content of 5 g granules was determined using a moisture analyzer (HC 103, Mettler-Toledo, Zaventem, Belgium) at 105 °C until the weight was constant for 30 s. Specific mechanical energy (SME) (kJ/kg) values were calculated (Equation (1)) based on the torque (*T,* Nm), throughput (*Q*, kg/min), and screw speed (*N*, rpm) to obtain a scale-independent parameter translating the energy introduced into the system applicable to multiple scales of twin screw granulators:

(1) $$\;\mathrm{SME}=\frac{T*N}{Q*1000}$$

Additionally, granulation experiments with MCC (95% *w*/*w*) and PVA 4-88 or PVA 18-88 (5% *w*/*w*) were performed at L/S-ratios of 0.20 and 0.60. The screw speed was set at 500 rpm for PVA 4-88 and at 700 rpm for PVA 18-88. The other granulation parameters were similar to those described in this section. The data of the granulation experiments with binders PVA 4-88, SOS CO 01, PVP K90 and HPMC E15 were used from previous studies by Vandevivere et al. [14,15].

### 2.5. Preparation and Fluidized Bed Drying of Acetaminophen-Containing Granules

APAP formulations were granulated and fluidized bed dried on the ConsiGma^TM^-1 system. This laboratory-scale equipment consists of an identical twin screw granulator unit as the C25; however, it has only a single dryer cell compared to six cells in the C25 system. The preblend consisting of paracetamol (50% *w*/*w*), mannitol (45% *w*/*w*), and the binder (5% *w*/*w*) was prepared in a 20 L tumbling mixer (Inversina Bioengineering, Wald, Switzerland). The loss-in-weight feeder (Brabender DDSR20, Duisburg, Germany) fed the preblend into the granulation unit at a throughput of 20 kg/h. The same screw configuration was used as described in Section 2.4. Demineralized water as granulation liquid was added through 1.6 mm nozzles connected to two out-of-phase peristaltic pumps (Watson Marlow, Cornwall, UK). The flow of the granulation liquid was controlled by a mass flow meter. The screw speed was set at 700 rpm. First, granulation experiments were performed to determine at which L/S-ratio granules with similar quality (friability < 8% and d50 of 2000 μm) were obtained for the different PVA grades. Based on these experiments, the L/S-ratio was set at 0.10, 0.10, and 0.11 for PVA 4-88, PVA 18-88, and PVA 40-88, respectively (Table 1). The cell filling time, inlet airflow and drying air temperature were set at 120 s, 72 m^3^/h and 50 °C, respectively. The data of the granulation and drying experiments with binders SOS CO 01 and HPMC E15 were used from a previous study by Vandevivere et al. [16].

### 2.6. Preparation of Tablets

Prior to tableting, APAP granules produced at the longest drying time were milled through a grater screen of 1500 μm at 900 rpm using the Quadro comil (U10, Quadro, ON, Canada) integrated in the C25-system (GEA Pharma Systems, Wommelgem, Belgium). The milled granules were blended with 2% magnesium stearate for 5 min at 49 rpm in a tumbling blender (T2F, W. A. Bachofen, Basel, Switzerland). The blend was tableted on a compaction simulator (STYL’One Evolution, Medelpharm, Beynost, France) equipped with one pair of flat-faced Euro B punches of 10 mm diameter (Natoli Engineering Company, Saint Charles, MO, USA). Compression tests were performed at main compaction pressures of 64, 127, 191, 255, 318, and 446 MPa with a targeted tablet weight of 400 mg. The tableting data of binders SOS CO 01 and HPMC E15 were used from a previous study by Vandevivere et al. [16].

### 2.7. Evaluation of Granules

#### 2.7.1. Loss-On-Drying

The residual moisture content of the APAP granules was determined in triplicate through LOD using a moisture analyzer (Mettler HC103, Mettler-Toledo, Zaventem, Belgium). A sample of 5 g was dried at 105 °C until the weight was constant for 30 s.

#### 2.7.2. Friability

The granule friability was determined in triplicate using a friabilator (PTF 300 Pharma Test, Hainburg, Germany). Before the test, the granule fraction < 250 μm was removed. A representative sample of 10 g granules (*I_wt_*) with 200 glass beads (mean diameter 4 mm) was placed in a drum rotating at a speed of 25 rpm for 10 min, subjecting the granules to falling shocks. Afterwards, the glass beads were removed, and the mass retained on a 250 μm sieve (*F_wt_*) was determined. The friability was calculated as $[\left(I_{wt}-F_{wt}\right)/I_{wt}]\;*\;100$.

#### 2.7.3. Particle Size Analysis

The granule size distributions were determined via dynamic image analysis using the QICPIC^TM^ system (Sympatec, Clausthal-Zellerfeld, Germany), equipped with a vibrating feeder system (Vibri/L^TM^) for gravimetric feeding of the granules. Ten sub-samples of the collected granules were obtained using a rotary cone sample divider (Laborette 27, Fritsch, Idar-Oberstein, Germany ), and three of these sub-samples were measured. The granule size (expressed as the diameter of a circle of equal projection area) was calculated using WINDOX 5 software (Sympatec, Clausthal-Zellerfeld, Germany).

### 2.8. Evaluation of Tablets

#### Tensile Strength Analysis

The hardness, thickness, and diameter of the tablets (n = 10) was determined using a hardness tester (ST50, Sotax, Saint-Louis, France). The tensile strength (TS) of the tablets was calculated according to Equation (2) described by Fell and Newton [23]:

(2) $$TS=\frac{2F}{\pi dt}$$

where *F*, *d* and *t* denote the diametral crushing force, tablet diameter, and tablet thickness, respectively. Furthermore, disintegration and friability testing was performed on tablets compressed at 255 MPa. Six tablets were tested in a disintegration bath (DT50, Saint-Louis, France) containing 800 mL of demineralized water at 37 °C. Friability was tested according to the European Pharmacopeia using a friabilator (PTF 300 Pharma Test, Hainburg, Germany) at a speed of 25 rpm for 4 min. The percentage weight loss was expressed as tablet friability.

## 3. Results

### 3.1. Binder Characterization

#### 3.1.1. Clustering of Binders According to Their Attributes

**Figure 2:**
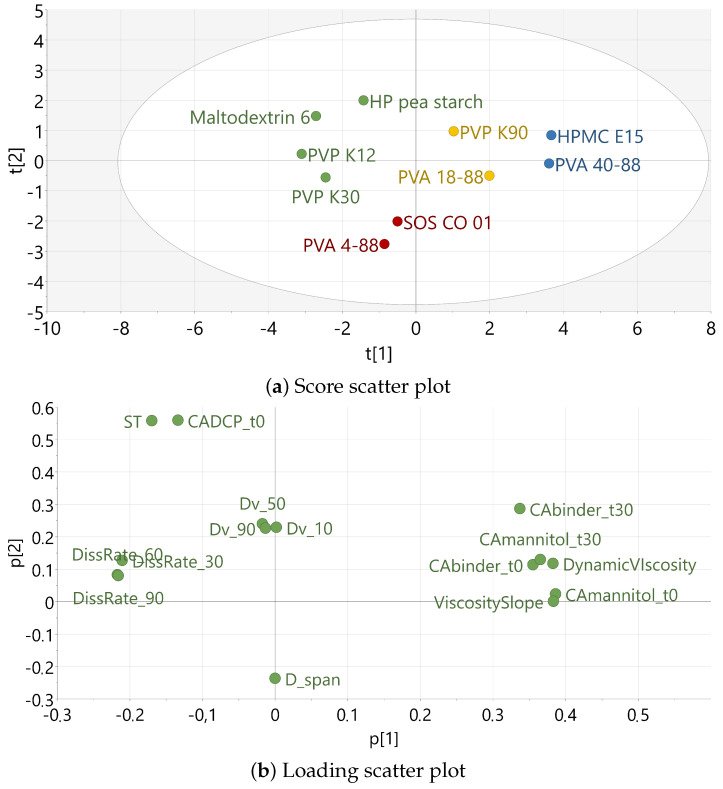
The score (**a**) and loading (**b**) scatter plot of PC 1 versus PC 2. The different colors refer to the binder clusters, which have comparable binder attributes. Cluster 1 (red) contains PVA 4-88 and SOS CO01; cluster 2 (yellow) has PVA 18-88 and PVP K90; cluster 3 (blue) has PVA 40-88 and HPMC E15; and cluster 4 (green) has maltodextrin 6, HP pea starch, PVP K12, and PVP K30.

The binder attributes of the three PVA grades were combined with the binder dataset of Vandevivere et al., and PCA was applied on the fifteen attributes of the binders in scope (Table A1) [14,15]. A model was built with two principal components (PCs), explaining, respectively, 62.8% and 22.8% of the variation, and hierarchical cluster analysis was applied to define the binder clusters. The score scatter plot (Figure 2a) reveals how the binders correlate to each other according to their principal properties. The loading scatter plot (Figure 2b) reveals the importance of a binder property towards its principal component and positive or negative correlations between the binder properties. Four binder clusters could be identified based on the binder attributes, each indicated with a specific color (Figure 2a). Cluster 1 (red) contains the binders PVA 4-88 and SOS CO 01. Based on the score and loading scatter plots, the binders in cluster 1 exhibit medium dissolution kinetics, medium binder wetting by water, medium wetting of hydrophilic surfaces, good wetting of hydrophobic surfaces, and medium viscosity. Cluster 2 (yellow) contains PVA 18-88 and PVP K90 which exhibit medium values for all characteristics and are consequently located close to the origin of the scores plot. Cluster 3 (blue) contains PVA 40-88 and HPMC E15, which are characterized by slow dissolution kinetics, poor binder wetting by water, poor wetting of hydrophilic surfaces, medium wetting of hydrophobic surfaces, and high viscosity. Cluster 4 (green) groups maltodextrin 6, HP pea starch and PVP K12 according to their characteristics but contains no PVA grade. Therefore, the cluster 4 binders were not further investigated in the current study. Although the investigated PVA grades (4-88, 18-88 and 40-88) differ solely in DP according to their nomenclature, this parameter dictates different characteristics: it has a direct impact on the viscosity but also indirectly impacts the dissolution rate and wettability.

#### 3.1.2. Surface Energy

**Figure 3:**
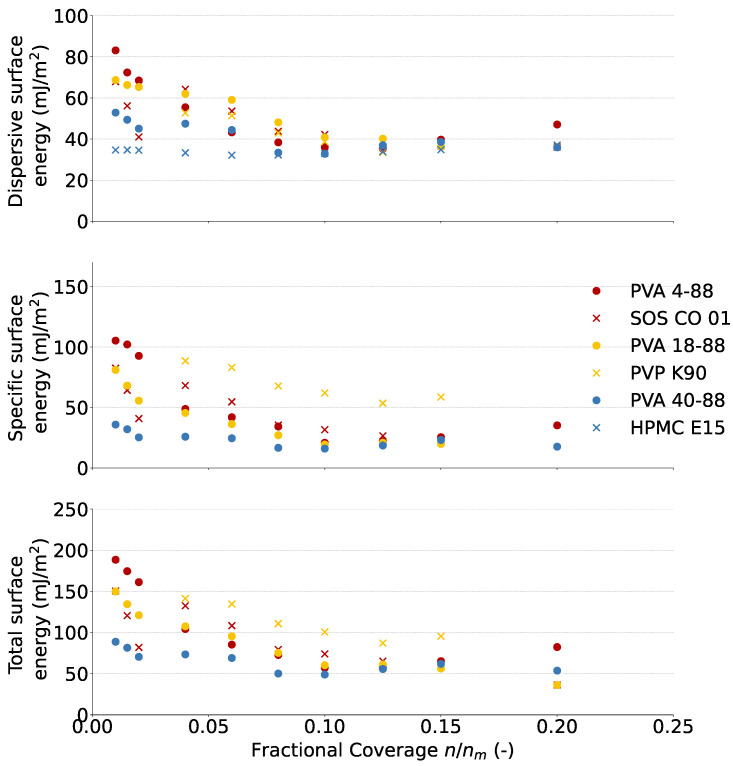
The dispersive (**top**), specific (**middle**) and total (**bottom**) surface energy in the function of the fractional coverage for cluster 1, 2, and 3 binders. The specific and total surface energies of HPMC E15 could not be measured due to weak solvent interactions.

Figure 3 shows the dispersive, specific, and total surface energy of the binders as a function of the surface coverage. The surface energy data were not included in the PCA plot, as they must be analyzed as a distribution function rather than a single value at a specific surface coverage.

The specific surface energy is a measure of specific or polar interactions such as hydrogen bonds. All binders showed surface energy heterogeneity, as the distribution function exponentially decreased in the function of coverage, indicating that they contained high-energy sites for polar surfaces. PVA 4-88 (105.29 mJ/m^2^) had the highest specific surface energy at coverage of 0.01 of all PVA grades, followed by PVA 18-88 (81.10 mJ/m^2^) and PVA 40-88 (35.86 mJ/m^2^), but at surface coverages of 0.10 or higher, their specific surface energies were similar. Therefore, at low surface coverages, PVA 4-88 will preferentially interact with polar surfaces compared to the other PVA grades. The contact angle measurements yielded comparable results as the specific surface energy measurements between the PVA grades. Although both measurements are assumed to give similar information about the powder surface, additional information is obtained by performing surface energy measurements. The contact angle is an average parameter measured over all sites on a powder surface at complete surface coverage, whereas the surface energy distribution gives information of the surface energy heterogeneity of the particle surface due to fractional coverage [24,25,26]. PVA 4-88 (45.85° ± 2.08 and 33.18° ± 2.90) had the lowest contact angle on the mannitol surface after 0 and 30 s, followed by PVA 18-88 (65.93° ± 1.24 and 40.43° ± 2.47) and PVA 40-88 (76.35° ± 3.36 and 45.63° ± 3.07), highlighting the better wetting of specific surfaces by PVA 4-88. At low surface coverage, PVA 4-88 and SOS CO 01 both contained high-energy sites, but those of PVA 4-88 had a higher specific surface energy than SOS CO 01. Agglomeration of the high-energy sites with polar surfaces will therefore be more likely achieved with PVA 4-88 compared to SOS CO 01. The specific surface energy of PVP K90 was not measurable at the low fractional coverages due to the sensitivity of the method. The specific surface energy of HPMC E15 was not measurable at all fractional coverages due to the noise on the signal; hence, very weak bonds were formed with the solvents. At a fractional coverage of 0.10, PVP K90 had a higher specific surface energy than the other binders, whose specific surface energies were similar at a coverage of 0.10 or higher. PVP K90 will therefore favorably bind with polar surfaces during agglomeration compared to the other binders.

The dispersive surface energy is a measure for the non-specific or apolar interactions due to van der Waals forces. All binders, except HPMC E15, showed surface energy heterogeneity, with high-energy sites for apolar surfaces. Comparing the PVA grades, the same trend as with the specific surface energy was observed: PVA 4-88 (83.14 mJ/m^2^) had the highest dispersive surface energy at coverage of 0.01 of all PVA grades, followed by PVA 18-88 (68.70 mJ/m^2^) and PVA 40-88 (52.85 mJ/m^2^), but at surface coverages of 0.125 or higher, their dispersive surface energies were similar. Therefore, at low surface coverages, PVA 4-88 will preferentially interact with apolar surfaces compared to the other PVA grades. Again, the ranking of the dispersive surface energies correlated with the ranking of the contact angle of the binder solution on the DCP surface. PVA 4-88 (34.57° ± 1.43) had the lowest contact angle on the DCP surface, followed by PVA 18-88 (68.77° ± 1.10) and PVA 40-88 (73.43° ± 2.62), highlighting the better wetting of dispersive surfaces by PVA 4-88. However, it should be noted that DCP still offers polar surfaces/interaction potential next to apolar surfaces. As the ranking of the contact angle measurements of the PVA grades on mannitol or DCP surface is similar, this interpretation can be retained. At low surface coverage, PVA 4-88 contained high-energy sites with a higher dispersive energy than those of SOS CO 01. The high-energy sites of PVA 4-88 are therefore more likely to agglomerate with apolar surfaces than SOS CO 01 at low coverages. The dispersive component of PVP K90 was not measurable at low surface coverage due to sensitivity issues, making a comparison with PVA 18-88 in the same binder cluster impossible. HPMC E15 lacked high-energy sites and showed a homogeneous surface energy irrespective of the coverage. Therefore, PVA 40-88 will favorably form bonds with apolar surfaces compared to HPMC E15. At an average fractional coverage of 0.10, no relevant differences were seen between the binders, indicating that the binders are equally likely to form interactions [24,26].

The dispersive and specific surface energies were combined into the total surface energy. Due to the larger contribution of the specific component to the total surface energy, it can be concluded that PVP K90 had the highest total surface energy at a surface coverage above 0.05. This means that its heterogeneous surface has good properties to agglomerate with polar and apolar surfaces during granulation. However, at low fractional coverage, PVA 4-88 had high-energy sites with the highest total surface energy. Therefore, these sites on the heterogeneous surface of PVA 4-88 will also favorably bind with polar and apolar surfaces during the agglomeration of particles.

### 3.2. Evaluation of the Robustness of Granulation Process

**Figure 4:**
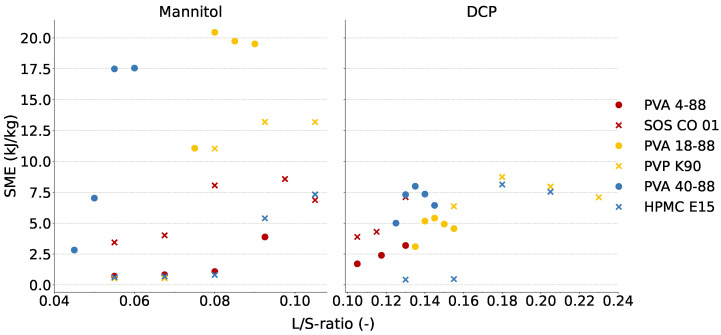
SME values in function of the L/S-ratio for each binder in mannitol- and DCP-based formulations. L/S-ratios depicted are based on the L/S-ratio range from Table 1.

Figure 4 shows the SME values in the function of the L/S-ratio for each binder in the water-soluble (mannitol) and water-insoluble (DCP) formulation. In the mannitol-based formulation, high SME values were obtained for PVA 18-88 and PVA 40-88 at the highest L/S-ratios of their respective L/S-ratio range. However, the SME remained substantially lower than the maximal SME (18 kJ/kg and 30 kJ/kg for DCP and mannitol-based formulations, respectively) tolerated by the granulator. Although PVP K90 and HPMC E15 were in the same binder cluster as PVA 18-88 and PVA 40-88, respectively, lower SME values were obtained for the former binders at the highest tested L/S-ratios. This implies that the high SME values obtained for both PVA grades cannot fully be attributed to the viscosity and/or wettability of the binders. In the DCP-based formulation, the SME was lower for each PVA grade compared to the mannitol-based formulation; this can be attributed to the contribution of water-soluble mannitol in the granulation process, whereas water-insoluble DCP will not contribute to bond formation.

Overall, the workable L/S-ratio range in both formulations was rather small when including the PVA grades compared to the other binder in the corresponding cluster (Table 1). In addition, a sudden increase in SME was obtained including PVA 18-88 and PVA 40-88 when the L/S-ratio was increased by 0.005 units L/S (which corresponds to 1.7 g/min granulation liquid). This limits the robustness of the granulation process, as a small amount of granulation liquid significantly affects the process and possibly the granule quality. To improve the robustness of the process, the commonly used filler MCC could be (partially) included in the formulation. MCC absorbs water, making the formulation less sensitive to overwetting due to an excess of granulation liquid [27]. The granulation experiments performed with formulations containing 95% *w*/*w* MCC and 5% *w*/*w* PVA (grade 4-88 or 18-88) clearly showed that the L/S-ratio range which allowed processing broadened, as granules with good properties could be obtained from a L/S-ratio of 0.20 to 0.60. The friability of MCC-based granules with PVA 4-88 was 14.8 ± 0.7% and 7.5 ± 0.4% at the lowest and highest L/S-ratios, respectively, for the PVA 18-88 grade; these values were 35.0% ± 2.4% and 7.1% ± 1.8%.

The narrow L/S-ratio range was inherent to the PVA grades, and it is hypothesized that the thermodynamics of PVA when granulated with water could explain this observation. PVA is a polymer with highly polar OH groups which form inter- and intramolecular hydrogen bonds (H-bonds). However, as water is only a moderate solvent for PVA from a thermodynamic perspective, the H-bonds formed between the interacting segments of PVA will be stronger than the H-bonds formed between the OH groups of PVA and water [28,29]. At a low L/S-ratio, the PVA polymer chain will have an isolated coil-like structure (based on the intramolecular H-bonds) due to the poor affinity for the solvent, without a significant increase in viscosity [28]. In contrast, at a higher L/S-ratio, the chain aggregation and gelation of PVA can occur when the PVA coils disentangle, forming intermolecular interactions with other PVA polymer chains and with water. Hence, the viscosity increases as the hydrodynamic friction and entanglement of PVA chains enhances the friction to flow [28]. This higher viscosity facilitates a sudden granule growth, explaining the narrow workable L/S-ratio range when processing formulations with PVA as binder. In addition, the SME increased due to the higher viscosity (Figure 4), with a sudden SME increase observed for PVA 18-88 and PVA 40-88 when changing from low to high L/S-ratios. Local higher temperatures in the granulator barrel due to the increased SME values could potentially also contribute to the breakage of intramolecular PVA bonds. However, as the granulator barrel is actively cooled, this increase in product temperature could not be directly measured.

### 3.3. Binder Effectiveness during Granulation of Water-Soluble and Water-Insoluble Formulations

#### 3.3.1. PVA Grades

**Figure 5:**
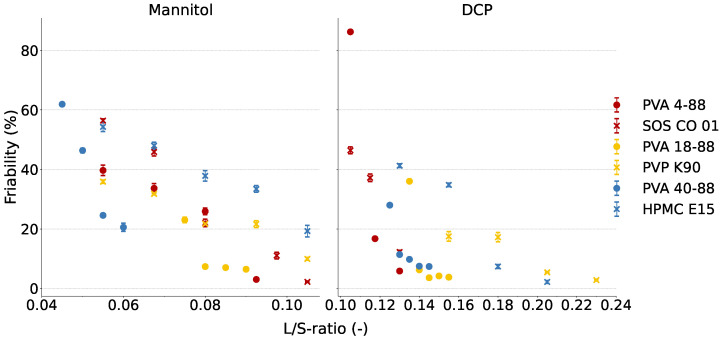
Friability in function of the L/S-ratio for each binder in the mannitol- and DCP-based formulations. The L/S-ratios depicted are based on the L/S-ratio range from Table 1.

The friability in the function of the L/S-ratio for the mannitol- and DCP-based formulation with different binders is shown in Figure 5. PVA 40-88 was the most effective PVA binder in the mannitol-based formulation, as it produced the strongest granules at the lowest L/S-ratio. However, the high SME values (Section 3.2) could limit its application during granulation. A pure mannitol-based formulation produced granules with the lowest friability of approximately 20% independent of the L/S-ratio [15]. Therefore, PVA 4-88 and PVA 18-88 were the only PVA grades in the mannitol-based formulation yielding a lower granule friability than pure filler. In the water-insoluble formulation, PVA 4-88 was the most effective PVA binder. At the highest L/S-ratio of their respective L/S-ratio range, the friability was comparable between the different PVA grades. During formulation development in continuous manufacturing, the consideration must be made between obtaining granules that are as strong as possible and a L/S-ratio that is as low as possible in order to rapidly dry the granules during downstream processing. Next, the process must be robust, and SME values should be evaluated. Therefore, PVA 4-88 was the preferred binder regardless of the solubility of the formulation.

It was difficult to unambiguously link the attributes of the PVA grades to their binder effectiveness in the water-soluble and water-insoluble formulations. According to the study of Vandevivere et al. [15], an appropriate binder for a good water-soluble formulation should have fast binder dissolution kinetics, low-viscosity attributes, and good wetting of the highly soluble formulation. For a water-insoluble formulation, the binder should have slow binder dissolution kinetics, high viscosity attributes and good wetting properties of dispersive surfaces. Studying the PVA grades, a different correlation between the binder attributes and binder effectiveness in function of the formulation solubility was seen compared to the study of Vandevivere et al. [15]. In the current study, the best binder for a good water-soluble formulation had slow binder dissolution kinetics and high viscosity attributes, while the optimal binder for the water-insoluble formulation had fast binder dissolution kinetics and low viscosity attributes. However, it should be noted that in the DCP-based formulation, the differences in the L/S-ratio between PVA 4-88 and PVA 40-88 were rather small for obtaining granules with friability below 30%.

PVA 18-88 was the least effective PVA binder for DCP-based formulation, as it needed the highest L/S-ratio to obtain high-quality granules. Although its binder attributes (viscosity, binder wetting, wetting of hydrophilic and hydrophobic surfaces, and dissolution kinetics) and total surface energy were between those of PVA 4-88 and PVA 40-88 (Section 3.1), this was not reflected in the required L/S-ratio. The PCA plot and surface energy measurements must be carefully interpreted to study binder effectiveness, as the granule growth can be influenced by an interplay of binder characteristics or a specific binder characteristic dominating the granulation process. Thielmann et al. [30] reported that a ’better wettability does not necessarily mean better granulation’. Collision between particles resulting in granule growth can be achieved if the wetted region of the particles comes into contact with each other and if the kinetic energy of the collision can be dissipated. On the one hand, if a powder with good wettability or high surface energy (e.g., PVA 4-88) is used during granulation, a thin layer of the granulation liquid will be coated on the particle’s surface, causing complete wetting. This increases the probability of merging if particles are hitting each other; however, it limits the amount of kinetic energy of the impact that can be dissipated due to its thinner layer. On the other hand, if a poorly wettable or powder with low surface energy (e.g., PVA 40-88) is used, the granulation liquid will only partially cover the particle’s surface more thickly, whereby the surface is partially covered. This limits the probability of merging if particles are hitting each other but increases the ability to dissipate the kinetic energy of the impact. As PVA 18-88 has medium viscosity and wettability compared to grades PVA 4-88 and PVA 40-88, it is therefore hypothesized that the agglomeration between particles including PVA 18-88 will be less likely. Therefore, more liquid was required to yield granules of similarly equal quality.

#### 3.3.2. Corresponding Cluster Binders

Table 2 and Figure 5 compare the effectiveness of the different binders included in the mannitol- and DCP-based formulations based on the L/S-ratio required to achieve granules with friability below 30% and the lowest achievable friability independent of the L/S-ratio. For the cluster 1 binders in the mannitol-based formulation, no difference was seen in the L/S-ratio required to yield granules with friability below 30%, and a comparable absolute friability was obtained. In the DCP-based formulation, PVA 4-88 was more effective than SOS CO 01, as PVA 4-88 yielded strong granules at a lower L/S-ratio, as well as stronger granules independently of the L/S-ratio. For the cluster 2 binders in the mannitol-based formulation, limited differences were seen in the binder effectiveness. In the DCP-based formulation, PVA 18-88 was more effective than PVP K90, as the former yielded strong granules at a lower L/S-ratio. In cluster 3, PVA 40-88 was significantly more effective than HPMC E15 in terms of the L/S-ratio for both formulations. However, at a higher L/S-ratio, HPMC E15 yielded equally strong granules as PVA 40-88 in the mannitol-based formulation and slightly stronger granules in the DCP-based formulation. The pronounced differences in binder effectiveness of the cluster 3 binders could be attributed due to the surface energy. As discussed in Section 3.1.2, HPMC E15 had a homogeneous dispersive surface energy, while PVA 40-88 had a heterogeneous dispersive surface energy with high-energy sites having a higher dispersive surface energy. Therefore, HPMC E15 is less likely to form apolar bonds during particle agglomeration than PVA 40-88. The specific surface energy of HPMC E15 is not measurable, as it forms weak bonds with polar solvents. This also explains why more granulation liquid has to be added when HPMC E15 is used as binder in the mannitol-based formulation.

Overall, it could be concluded that for the water-insoluble formulation, the PVA grades were more effective than the other binder in the corresponding cluster. Limited differences were observed between the binders of clusters 1 and 2 for the good water-soluble formulation, whereas in cluster 3, PVA 40-88 was more effective than HPMC E15. Binder addition is essential in a water-insoluble formulation, as solely the binder is responsible for bond formation between the particles [15]. The beneficial effect of the PVA grades compared to other binders in their respective cluster for the DCP-based formulation could be attributed to two binder attributes. First, the PVA grades had a higher dispersive surface energy at low fractional coverage than their corresponding cluster binders, indicating the favorable formation of apolar bonds at its high-energy sites. Second, PVA grades had a significantly lower surface tension compared to PVP K90 and HPMC E15 (Table A1). The lower liquid surface tension reduces the capillary suction pressure, which in turn reduces the frictional resistance to particle rearrangement. The PVA particles will dissolve in water during granulation, which lowers the surface tension of the granulation liquid and increases the consolidation rate of the particles [31].

The clustering of binders according to their binder attributes proved valuable; however, the clustering did not fully align with the granulation behavior. The binders included in clusters 1 and 2 show comparable behavior in terms of the friability and needed L/S-ratio, whereas the behavior of the binders in cluster 3 is less similar. The differences are partially attributed to the differences in surface energy data between these binders. These data are not included in the PCA plot because of missing data and the lack of descriptors describing the distribution of the surface energy data as a function of the surface coverage (Section 3.1.2). To aid future formulation development, the surface energy data should be interpreted next to the characterization data included in the PCA plot.

### 3.4. Binder Effectiveness during Fluidized Bed Drying

Next to studying the binder effectiveness in a water-soluble and water-insoluble formulation, the binder effectiveness during granulation and the drying and breakage behavior during the fluidized bed drying of granules containing a poorly water-soluble drug (APAP) was studied. Granules with similar quality (d50 of 2000 μm and friability below 8%) were produced. The L/S-ratios to obtain the predetermined granule quality are listed in Table 3. A comparison was made of the cluster 1 and 3 binders SOS CO 01 and HPMC E15 from the study of Vandevivere et al. [16]. At the mentioned L/S-ratio, those binders had lower d50 values than the PVA grades but met the friability criterion (Table 3). Due to the limited process robustness of the PVA grades, smaller granules which complied with the friability criterion could not be obtained.

#### 3.4.1. Drying Behavior

**Figure 6:**
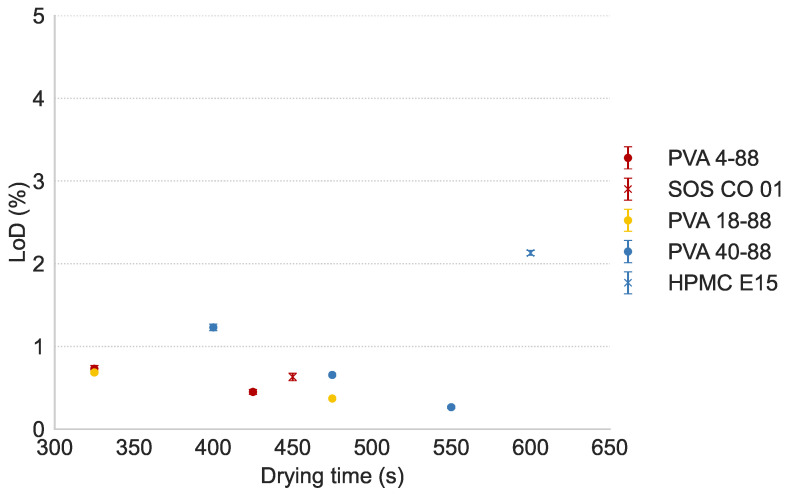
Moisture content of the granules in function of drying time. Granules were produced on the L/S-ratio mentioned in Table 3.

Figure 6 shows the moisture content of the APAP granules in the function of the drying time. The PVA grades were dry (LOD < 1%) within a reasonable time period, namely, 325 s for PVA 4-88 and PVA 18-88 and 475 s for PVA 40-88. As reported in the study of Vandevivere et al. [16], the drying time was linked with the L/S-ratio needed for granulation. The drying efficiency of cluster 1 binders (PVA 4-88 and SOS CO 01) was comparable. In contrast, in cluster 3, PVA 40-88 was more effective in terms of drying efficiency than HPMC E15 due to the lower L/S-ratio required for granulation with PVA 40-88. This was also previously observed in the water-soluble and water-insoluble formulations and emphasizes the importance of binder effectiveness at a low L/S-ratio, as it results in a shorter run time.

#### 3.4.2. Granule Breakage

**Figure 7:**
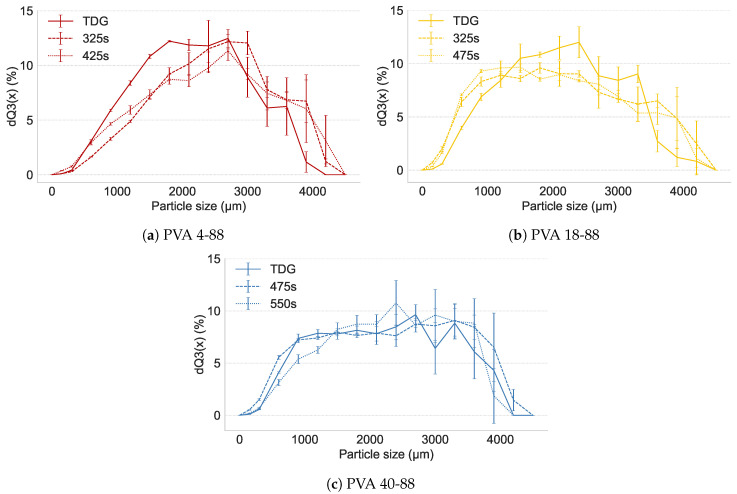
Breakage behavior of APAP granules with PVA grades. Solid line represents the particle size distribution of tray-dried granules (TDG). Dashed and dotted lines represent the particle size distribution after fluid bed drying at first and second drying times, respectively.

Figure 7a shows the particle size distribution of PVA 4-88 granules after tray-drying (TD) and FBD at two drying times. APAP granules containing PVA 4-88 showed no breakage during FBD and no significant differences in PSD between the drying times. The granules dried through FBD even showed an increase in particle size distribution compared to TD, which is physically not possible. This could be attributed to the limited process robustness with PVA with a small change in liquid inducing a relatively large increase in PSD of the granules (Section 3.2). PVA 18-88 granules showed limited breakage during FBD, but no attrition occurred, as the fines fraction did not increase. PVA 40-88 granules showed no breakage during FBD. It could be concluded that all PVA grades were suitable for FBD. However, formulation adjustments should be made to ensure a robust granulation process (e.g., the addition of MCC as reported in Section 3.2).

### 3.5. Impact of Binder Properties on Tableting Behavior

**Figure 8:**
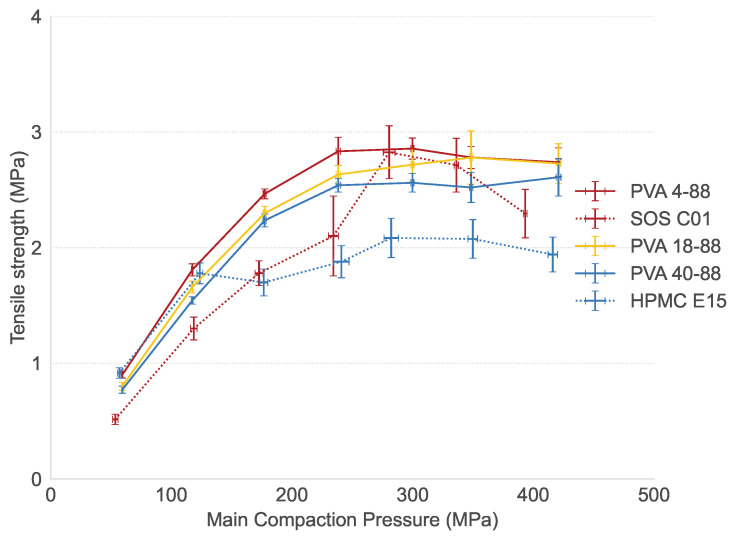
Tensile strength in function of main compaction pressure for studied binders in APAP formulation.

All PVA grades produced strong tablets (tensile strength > 1.7 MPa) using a compaction pressure of at least 191 MPa (Figure 8). The distinct binder characteristics between the PVA grades did not impact tabletability. All tablets met the European Pharmacopoeia criterion of a friability lower than 1% [32]. PVA 4-88 and 40-88 yielded stronger tablets at a lower compaction pressure than SOS CO 01 and HPMC E15, respectively.

## 4. Conclusions

The performance of three PVA grades during TSWG was investigated in depth and compared to binders with similar characteristics based on PCA of a wide variety of binders. All PVA grades produced high-quality granules at a low L/S-ratioS. PVA 4-88 was the most effective binder of the PVA grades for good water-soluble (mannitol) and water-insoluble (DCP) formulations. This was related to its high total surface energy, low viscosity, good wettability of hydrophilic and hydrophobic surfaces, and good wettability by water compared to the other PVA binders. However, better binder wettability does not always yield better agglomeration, as PVA 40-88 was more effective than PVA 18-88 despite the latter’s better wettability. This was attributed to the high viscosity of PVA 40-88 which favors agglomeration when colliding. The PVA grades required granulation in a narrow L/S-ratio range, which limited the process robustness in DCP- and mannitol-based formulations. However, the inclusion of a water-absorbing component, such as MCC, broadened the L/S-ratio range. Compared to binders with similar properties in terms of PSD, viscosity, wettability, and solubility rate, the PVA grades proved especially favorable to include in a water-insoluble formulation, in which the binder has more impact than in a good water-soluble formulation. This was attributed to the high dispersive surface energy of the high-energy sites and low surface tension of the PVA grades. The drying efficiency of the binders was linked with the L/S-ratio at which the APAP granules with PVA grades were produced, allowing short drying times due to the production of granules at low L/S ratios. The APAP granules showed no breakage and yielded strong tablets with low friability, irrespective of the PVA grade. PVA could be the preferred binder in TSWG due to the low L/S-ratio at which high-quality granules are produced, as high L/S ratios could limit the throughput of a continuous TSWG line by requiring long drying times. However, attention needs to be given to the robustness of the granulation process.

## Figures and Tables

**Table 1:** L/S-ratios for granulation experiments of the good water-soluble (mannitol) and insoluble (DCP) formulations containing the different binders on the C25 system. L/S-ratio resulting in similar granule quality (friability < 8% and d50 of 2000 μm) and set drying time during fluidized bed drying of the model drug formulations on the C1 system.

Binder	C25		C1	
	**Mannitol Formulation**	**DCP Formulation**	**Model Drug Formulation**	
	**L/S-Ratio Range**	**L/S-Ratio Range**	**L/S-Ratio**	**Drying Time (s)**
PVA 4-88	0.0550–0.0925 *	0.1050–0.1300 *	0.1000	325; 425
SOS CO 01	0.0550–0.1050 *	0.1050–0.1300 *	0.1050 *	450 *
PVA 18-88	0.0750–0.0900	0.1350–0.1550	0.1000	325; 475
PVP K90	0.0550–0.1050 *	0.1550–0.2300 *	0.1200	550
PVA 40-88	0.0450–0.0600	0.1250–0.1450	0.1100	400; 475; 550
HPMC E15	0.0550–0.1050 *	0.1300–0.2050 *	0.1800 *	600 *

* Experiments were performed in previous studies [14,15,16].

**Table 2:** Comparison of the binder effectiveness of cluster binders 1, 2 and 3 for the mannitol- and DCP-based formulations. The L/S-ratio was the lowest L/S-ratio, for which the friability was below 30%. The friability (%) was the lowest achievable friability regardless of the L/S-ratio. SME (kJ/kg) was the SME value measured at the mentioned L/S-ratio.

Binder		Mannitol Formulation			DCP Formulation		
		**L/S-Ratio**	**Friability (%)**	**SME (kJ/kg)**	**L/S-Ratio**	**Friability (%)**	**SME (kJ/kg)**
Cluster 1	PVA 4-88	0.0800	3.03 (±0.26)	1.09	0.1175	5.85 (±0.35)	2.40
	SOS CO 01	0.0800	2.23 (±0.26)	8.05	0.1300	12.26 (±0.58)	7.11
Cluster 2	PVA 18-88	0.0750	6.45 (±0.12)	11.07	0.1400	3.79 (±0.17)	5.17
	PVP K90	0.0800	9.94 (±0.48)	11.04	0.1550	2.82 (±0.31)	6.37
Cluster 3	PVA 40-88	0.0550	20.57 (±1.38)	17.49	0.1250	7.38 (±0.08)	5.00
	HPMC E15	0.1050	19.29 (±1.94)	7.32	0.1800	2.19 (±0.51)	8.13

**Table 3:** L/S-ratios yielding granules with similar quality in terms of friability (<8%) and PSD (d50 ± 2000 μm).

Binder		Model Drug Formulation		
		**L/S-Ratio**	**Friability (%)**	**d50 (μm)**
Cluster 1	PVA 4-88	0.100	2.57 (±0.12)	2026.3 (±23.8)
	SOS CO 01	0.105	7.03 (±0.95)	1699.6 (±55.5)
Cluster 2	PVA 18-88	0.100	7.25 (±0.50)	2032.2 (±71.7)
Cluster 3	PVA 40-88	0.110	5.07 (±0.22)	2324.9 (±19.6)
	HPMC E15	0.180	0.77 (±0.05)	1529.0 (±13.8)

## Data Availability

Data are contained within the article.

## Appendix A. Binder Characterization

**Table A1:** Raw data of binder attributes. Binders with asterisk (*) were characterized in study of Vandevivere et al. [14,15], except CAmannitol_t0 and CAmannitol_t30 of PVP K30.

Binder	Dv_10 (μm)	Dv_50 (μm)	Dv_90 (μm)	D_span	DissRate_30 (%)	DissRate_60 (%)	DissRate_90 (%)	
**PVA 4-88**	9.6 (±0.2)	27.1 (±1.1)	93.8 (±1.1)	3.1 (±0.1)	3.7 (±0.0)	6.1 (±0.3)	7.3 (±0.0)	
**PVA 18-88**	18.5 (±0.3)	68.3 (±0.3)	121.5 (±2.6)	1.5 (±0.0)	2.2 (±0.0)	3.0 (±0.2)	4.6 (±0.3)	
**PVA 40-88**	19.5 (±0.1)	66.4 (±0.9)	168.8 (±4.2)	2.3 (±0.0)	1.5 (±0.3)	2.3 (±0.4)	4.3 (±0.3)	
**SOS CO 01 ***	14.6	95.5	212.3	2.1	3.8	6.1	6.1	
**Maltodextrin 6 ***	27.6 (±3.5)	123.8 (±2.4)	270.0 (±8.7)	2.0 (±0.1)	9.7 (±0.0)	10.0 (±0.0)	10.0 (±0.0)	
**HP pea starch ***	105.8 (±1.2)	216.9 (±2.9)	374.9 (±4.8)	1.2 (±0.0)	8.7 (±0.0)	10.0 (±0.0)	10.0 (±0.0)	
**PVP K12 ***	13.5 (±0.4)	49.3 (±1.0)	100.4 (±1.0)	1.8 (±0.0)	9.6 (±0.0)	9.9 (±0.0)	9.9 (±0.0)	
**PVP K30 ***	29.2 (±0.6)	83.0 (±0.2)	144.1 (±1.5)	1.4 (±0.0)	8.0 (±0.0)	9.5 (±0.0)	10.0 (±0.0)	
**PVP K90 ***	83.0 (±2.1)	173.0 (±4.7)	315.1 (±7.1)	1.3 (±0.0)	3.0 (±0.0)	4.0 (±0.0)	6.6 (±0.0)	
**HPMC E15 ***	38.6 (±0.3)	89.5 (±0.5)	177.7 (±2.0)	1.6 (±0.0)	0.5 (±0.0)	2.5 (±0.0)	2.5 (±0.0)	
**Binder**	**CAbinder_t0 (°)**	**CAbinder_t30 (°)**	**CAmannitol_t0 (°)**	**CAmannitol_t30 (°)**	**CADCP_t0 (°)**	**ST (mN/m)**	**Dynamic Viscosity (mPa.s)**	**Viscosity Slope**
**PVA 4-88**	62.9 (±3.1)	33.4 (±2.4)	45.9 (±2.1)	33.2 (±2.9)	34.6 (±1.4)	31.2 (±0.0)	8.3 (±0.2)	0.2
**PVA 18-88**	71.4 (±4.4)	63.6 (±3.1)	65.9 (±1.2)	40.4 (±2.5)	68.8 (±1.1)	33.8 (±0.1)	166.2 (±2.2)	0.2
**PVA 40-88**	75.5 (±1.8)	73.0 (±0.2)	76.4 (±3.4)	45.6 (±3.1)	73.4 (±2.6)	34.3 (±0.1)	574.9 (±6.4)	0.3
**SOS CO 01 ***	68.7	47.2	47.3	33.9	32.8	30.2	4.1	0.1
**Maltodextrin 6 ***	60.0 (±3.9)	47.8 (±2.5)	40.9 (±2.5)	35.4 (±7.8)	103.0 (±5.3)	66.8 (±0.2)	2.1 (±0.0)	0.1
**HP pea starch ***	67.0 (±3.8)	57.4 (±4.6)	43.7 (±0.5)	33.0 (±0.5)	95.3 (±0.5)	57.7 (±0.5)	15.7 (±0.5)	0.1
**PVP K12 ***	NA	NA	39.8 (±2.0)	26.9 (±1.5)	109.6 (±3.1)	50.7 (±0.5)	1.6 (±0.0)	0.0
**PVP K30 ***	66.9 (±3.2)	37.3 (±3.3)	40.3 (±2.0)	27.2 (±1.8)	81.3 (±2.9)	43.3 (±0.2)	4.3 (±0.1)	0.1
**PVP K90 ***	76.7 (±2.9)	56.0 (±1.0)	55.4 (±1.9)	38.0 (±0.9)	84.7 (±1.3)	44.7 (±0.4)	99.5 (±1.6)	0.2
**HPMC E15 ***	79.1 (±1.7)	70.2 (±1.8)	69.4 (±2.6)	51.3 (±0.9)	69.6 (±4.4)	49.0 (±0.2)	817.1 (±2.8)	0.3
